# MOMO Syndrome with Holoprosencephaly and Cryptorchidism: Expanding the Spectrum of the New Obesity Syndrome

**DOI:** 10.1155/2011/839650

**Published:** 2011-09-22

**Authors:** Sheetal Sharda, Inusha Panigrahi, Ram Kumar Marwaha

**Affiliations:** Genetic and Metabolic Unit, Department of Pediatrics, Advanced Pediatric Center (APC), Postgraduate Institute of Medical Education and Research (PGIMER), Chandigarh 160012, India

## Abstract

There are multiple genetic disorders with known or unknown etiology grouped under obesity syndromes. Inspite of having multisystem involvement and often having a characteristic presentation, the understanding of the genetic causes in the majority of these syndromes is still lacking. The common obesity syndromes are Bardet-Biedl, Prader-Willi, Alstrom, Albright's hereditary osteodystrophy, Carpenter, Rubinstein-Taybi, Fragile X, and Börjeson-Forssman-Lehman syndrome. The list is ever increasing as new syndromes are being added to it. One of the recent additions is MOMO syndrome, with about five such cases being reported in literature. Expanding the spectrum of clinical features, we report the first case of MOMO syndrome from India with lobar variant of holoprosencephaly and cryptorchidism, which have not been reported previously.

## 1. Case Report

A one-and-a-half-year-old male child was evaluated for global developmental delay and a large head size which was observed since birth. He was the only child of a nonconsanguineous couple. He was born to a multigravida mother with history of three previous first-trimester abortions. There was no history of treatment taken for recurrent spontaneous abortions. There was history of drug ingestion in the mother for low-grade fever and seizures during antenatal period (probably taken some alternative form of medicine). He was a full-term baby delivered vaginally with a low birth weight of 2000 gms but with no postnatal complications. A large head was observed right at birth which was progressive and had reached the present size of 50 cms.

**Figure 1:**
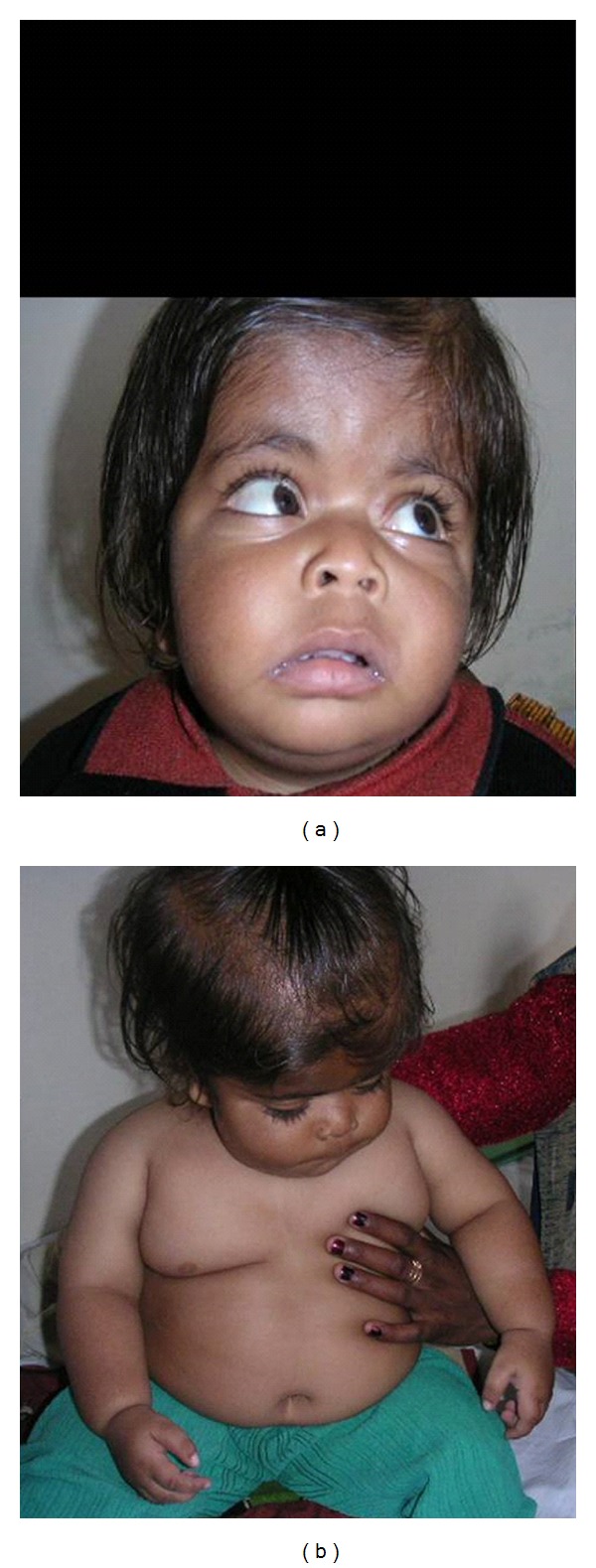
Clinical photograph showing facial dysmorphism and generalized obesity.

The child was admitted for bronchopneumonia at two months of age. The child had progressively gained weight over the previous eight months. The mother noticed decreasing vision over the past 2-3 months. Moreover, he had two episodes of seizures at 10 and 13 months of age. The child had global developmental delay. At 18 months, he had partial head holding and he was not able to sit or stand with support. He was not able to follow objects for the past 3 months but turned his head to sound. On general physical examination, coarse facial features, generalized obesity, horizontal nystagmus, and nonfollowing of light source were noted. Facial dysmorphism was in form of macrocephaly, downslanting palpebral fissures, broad depressed nasal bridge, flat nose, long philtrum, and thick lips (Figure 1). Anthropometric measurements revealed a weight of 15 kg (>95th percentile for age), a length of 77 cms (at 10th percentile for age), and a head circumference of 50 cms (>95th percentile for age). Systemic examination revealed hepatosplenomegaly with a liver span of 11 cms and spleen 2 cms below left costal margin. The child had bilateral undescended testis with empty scrotal sacs. Skin examination revealed multiple café-au-lait spots with the largest measuring approximately 9 × 8 cms. The child was initially investigated on the lines of storage disorder in view of macrocephaly, coarse facies, developmental delay, visual abnormality, and hepatosplenomegaly. 

**Figure 2:**
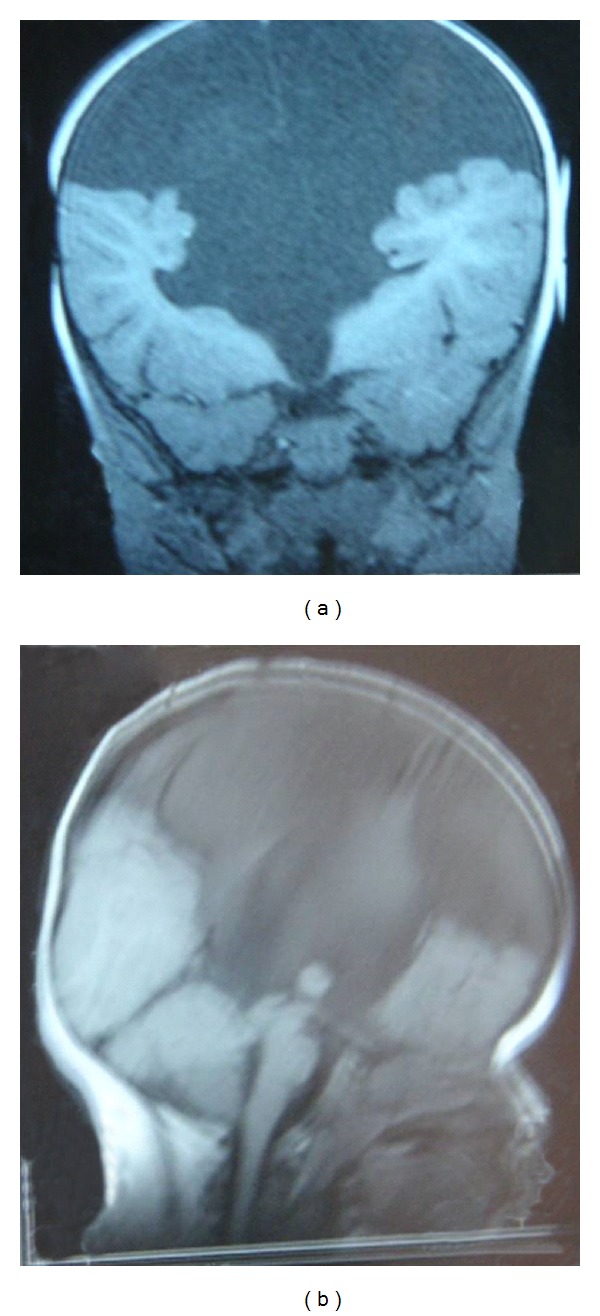
Magnetic resonance imaging of the brain revealing lobar holoprosencephaly.

Complete haemogram showed anemia with hypochromia and microcytosis with normal platelet count and normal leukocyte counts. The biochemical profiles including renal parameters were within normal range for age. Thyroid function tests were also within normal range. Urine spot test for mucopolysaccharidosis was negative on two occasions. Complete ophthalmic evaluation including fundus examination showed nonglaucomatous cupping of disc with right congenital disc anomaly suggestive of a coloboma. Chromosomes from peripheral blood showed 46, XY, normal male karyotype. Ultrasound of abdomen revealed raised liver echotexture with hepatomegaly. Bone marrow examination revealed no storage cells. Magnetic resonance imaging (MRI) of the pelvis showed undescended left testis and non visualized right testis. MRI brain was reported as “presence of CSF intensity fluid occupying part of supratentorial vault. Bilateral ventricles and third ventricle are not separately appreciated. Falx cerebri is intact and seen in midline and bilateral thalami are partially fused. Bilateral visualized part of frontal, parietal and occipital lobes and cerebellum is also normal.” The features were suggestive of lobar variant of holoprosencephaly.” (Figure 2).

## 2. Discussion

Acronym MOMO syndrome (MIM, 157980) stands for macrosomia, obesity, macrocrania, and ocular abnormalities with uncertain inheritance. Following our investigative findings, we considered the diagnosis of MOMO syndrome having all the four major components, that is, macrosomia, obesity, macrocrania and ocular abnormalities, with additional findings of cryptorchidism, hepatosplenomegaly, and lobar holoprosencephaly. Our case had many of the clinical findings reported in OMIM [2] and also few unreported features. 

The first two cases were reported in 1993 by Moretti-Ferreira et al. [3]. Both the cases had common clinical features consisting of truncal obesity, mental retardation, and ocular abnormalities. The facial features were relatively nonspecific with hypertelorism, downslanting palpebral fissures, a prominent forehead, and a broad nasal root. 

Later on more single case reports were added by Zannolli et al. in 2000 [4], Giunco et al. in 2008 [5], and the most recent addition by Wallerstein and Sugalski in 2010 [1]; thus expanding the spectrum of clinical manifestation in this syndrome. 

The first two cases described had height >90 percentile for age, but the third case reported by Zannoli et al. had short stature. Our case too has a length falling between 10 and 25th percentile of age. The clinical findings have now expanded based on these cases which include autism, short stature, clavicular pseudoarthrosis, and straight femurs to recurvation of the femur. Our case has holoprosencephaly, cryptorchidism, and hepatosplenomegaly as additional features (Table 1).

The etiology initially described in the first two cases was attributed to *de novo* autosomal mutation thus suggesting autosomal dominant inheritance, but till more cases are reported and more diagnostic tests are not carried out, it has been accepted as a syndrome of uncertain inheritance [6]. Description of more cases will help us elucidate the wide spectrum of clinical features and will also help us in finding clues to the obesogenic pathways. Obesity syndromes have variable clinical manifestation often involving multiple systems. Some may even have overlapping symptoms [5] which indicates a common pathway in the mechanism of obesity and thus can help us identify the molecular mechanisms involved in their etiology. Molecular basis of Bardet-Biedl syndrome has been recently identified, confirming it to be an oligogenic disorder. Prader-Willi syndrome is the commonest obesity syndrome seen in a genetic clinic or endocrinology clinic and has been proven to be due to loss of imprinted genes on 15q11-13. Other genetic obesity syndromes include Alstrom, Albright's hereditary osteodystrophy, Börjeson-Forssman-Lehman, Carpenter syndrome, Cohen, Fragile X, and Rubinstein-Taybi syndrome. Chromosomal abnormalities have also been associated and reported in obesity syndromes [7]. 

Due to lack of adequate literature from around the world and lack of investigative facilities, many of such obesity syndromes may go undiagnosed or unreported. Our case is possibly the first reported case from the Indian subcontinent. Moreover, the additional findings of holoprosencephaly and cryptorchidism add to the list of clinical features reported earlier.

## Figures and Tables

**Table 1:** Comparison of the cases reported in literature [1].

Cases reported	Moretti-Ferreira et al., [3]	Moretti-Ferreira et al., [3]	Zannoli et al., [4]	Wallerstein and Sugalski, [1]	Present case 2011
Sex	M	F	F	M	M
Macrosomia	+	+	−	+	+
Obesity	+	+	+	+	+
Macrocephaly	+	+	+	+	+
Ocular abnormalities	+	+	+	+	+
Downslanting palpebral fissures	+	+	+	+	+
Hypertelorism	+	+	+	+	+
Broad nasal root	+	+	+	+	+
Mental deficiency	+	+	+	+	+
High broad forehead	+	+	+	+	+
Delayed bone maturation	+	+	+	+	+
Additional features			Recurvation of the femur, Short stature	Sparse eyebrows, Clavicular pseudoarthrosis	Cryptorchidism, Holoprosencephaly, Hepato-splenomegaly
Thyroid studies	Normal	Normal	Normal	Normal	Normal
Karyotype					Normal
